# Supplementary material for: Polyketide synthase gene clusters in the ericoid mycorrhizal fungus Oidiodendron maius: genome-wide prediction, expression profiling, and functional characterization of knockout mutants
Source: Front Plant Sci. 2026 Sep 16;17:1924430. doi: 10.3389/fpls.2026.1924430 (PMC13623903; doi:10.3389/fpls.2026.1924430)
Supplement: Supplementary file 1 [file SupplementaryFile1.zip › Supplementary Materials/Supplementary Folder 1.zip!/Oidma1_AssemblyScaffolds_Repeatmasked/knownclusterblast/cluster8/jgi.p_Oidma1_23736_mibig_hits.html]

| MIBiG Protein | Description | MIBiG Cluster | MiBiG Product | % ID | % Coverage | BLAST Score | E-value |
| --- | --- | --- | --- | --- | --- | --- | --- |
| AGO65986.1 | putative\_monooxygenase | BGC0000992 | Fusaridione\_A\_biosynthetic\_gene\_cluster | 47 | 60.9677419355 | 170 | 2.5e-42 |
| ANY57883.1 | PenE | BGC0001372 | Penigequinolone\_biosynthetic\_gene\_cluster | 46 | 60.3225806452 | 157 | 2.9e-38 |
| EAU29811.1 | predicted\_protein | BGC0001400 | Citreoviridin\_biosynthetic\_gene\_cluster | 44 | 60.9677419355 | 154 | 2.5e-37 |
| AAT65719.1 | monooxygenase | BGC0000629 | Aflatrem\_biosynthetic\_gene\_cluster | 43 | 60.3225806452 | 153 | 4.2e-37 |
| AAW88511.1 | FAD\_dependent\_monooxygenase | BGC0001081 | Lolitrem\_biosynthetic\_gene\_cluster | 47 | 57.0967741935 | 149 | 7.9e-36 |
| CBF87267.1 | FAD-dependent\_monooxygenase,\_putative\_(AFU\_orthologue;\_AFUA\_8G02380) | BGC0000680\_c2 | Austinol/dehydroaustinol,\_locus\_1\_biosynthetic\_gene\_cluster | 47 | 56.7741935484 | 148 | 1e-35 |
| EAL89233.1 | FAD-dependent\_monooxygenase\_(PaxM),\_putative | BGC0000129 | Pyripyropene\_biosynthetic\_gene\_cluster | 43 | 65.1612903226 | 144 | 2.5e-34 |
| AAK11530.1 | PaxM | BGC0001082 | Paxilline\_biosynthetic\_gene\_cluster | 42 | 55.1612903226 | 140 | 3.7e-33 |
| BAM84049.1 | FAD-dependent\_monooxygenase | BGC0001260 | Terpendole\_E\_biosynthetic\_gene\_cluster | 40 | 60.6451612903 | 130 | 2.9e-30 |
| CCH26290.1 | putative\_FAD-dependent\_monooxygenase | BGC0001158 | Bikaverin\_biosynthetic\_gene\_cluster | 38 | 59.3548387097 | 120 | 3e-27 |
| ACZ57545.1 | predicted\_monooxygenase | BGC0000046 | Depudecin\_biosynthetic\_gene\_cluster | 37 | 60.6451612903 | 116 | 5.7e-26 |
| CBX87031.1 | BcBOA8\_protein | BGC0001243\_c2 | Botryenalol\_/\_botrydial\_/\_dihydrobotrydial\_/\_botryendial\_/ | 44 | 54.5161290323 | 114 | 1.6e-25 |
| CCT67992.1 | bikaverin\_cluster-monooxygenase | BGC0000030 | Bikaverin\_biosynthetic\_gene\_cluster | 35 | 59.3548387097 | 108 | 1.2e-23 |
| ACB47064.1 | FAD-dependent\_oxidoreductase | BGC0001060 | Dynemicin\_biosynthetic\_gene\_cluster | 33 | 54.8387096774 | 74 | 2.5e-13 |
| AAT65710.1 | putative\_monooxygenase | BGC0000629 | Aflatrem\_biosynthetic\_gene\_cluster | 29 | 56.7741935484 | 57 | 2.4e-08 |
| AMK51267.1 | Hex10 | BGC0001376 | Hexaricin\_biosynthetic\_gene\_cluster | 30 | 59.0322580645 | 52 | 1e-06 |
